# Description of a new species of Music frogs (Anura, Ranidae, *Nidirana*) from Mt Dayao, southern China

**DOI:** 10.3897/zookeys.858.34363

**Published:** 2019-07-01

**Authors:** Zhi-Tong Lyu, Yun-Ming Mo, Han Wan, Yu-Long Li, Hong Pang, Ying-Yong Wang

**Affiliations:** 1 State Key Laboratory of Biocontrol/ The Museum of Biology, School of Life Sciences, Sun Yat-sen University, Guangzhou 510275, China Sun Yat-sen University Guangzhou China; 2 Natural History Museum of Guangxi, Nanning 530012, China Natural History Museum of Guangxi Nanning China

**Keywords:** bioacoustic, Guangxi, mitochondrial DNA, morphology, *Nidiranayaoica* sp. nov.

## Abstract

A new species of Music frogs, *Nidiranayaoica***sp. nov.** is described based on a series of adult male specimens collected from Mt Dayao, Guangxi, southern China, providing valuable new information on the phylogeny, bioacoustics, and biogeography of related species within the genus *Nidirana*. The new species forms the sister taxon to *N.daunchina* from western China and together the sister taxon to *N.chapaensis* from northern Vietnam. *Nidiranayaoica***sp. nov.** can be distinguished from all known congeners by a significant genetic divergence in the mitochondrial 16S and CO1 genes, the advertisement call containing 1–3 rapidly repeated regular notes, and the combination of morphological characteristics including a medium-sized body with SVL 40.4–45.9 mm in adult males; lateroventral grooves on every digit, not meeting at the tip of disk; tibio-tarsal articulation reaching the nostril; the presence of a pair of subgular vocal sacs in males; and one single developed nuptial pad on dorsal surface of first finger in males.

## Introduction

The taxonomic treatment of the Music frog genus *Nidirana* Dubois, 1992 was controversial for a long time (Dubois 1987, 1992; Chen et al. 2005; Frost et al. 2006; Fei et al. 2009, 2010; Chuaynkern et al. 2010). The recent contribution to the phylogeny of genus *Nidirana* reconsidered it as a distinct genus, on the basis of comprehensive evidence of morphology, molecular phylogeny, bioacoustics, and biogeography (Lyu et al. 2017). Eight Music frog species were recognized from subtropical eastern and southeastern Asia (Lyu et al. 2017; Frost 2019): *N.okinavana* (Boettger, 1895) from Yaeyama of southern Ryukyu, and eastern Taiwan; *N.adenopleura* (Boulenger, 1909) from Taiwan and southeastern mainland China; *N.nankunensis* Lyu, Zeng, Wang, Lin, Liu & Wang, 2017 from Mt Nankun of Guangdong and *N.hainanensis* (Fei, Ye, & Jiang, 2007) from Mt Diaoluo of Hainan, both in southern China; *N.daunchina* (Chang, 1933) from western China; *N.pleuraden* (Boulenger, 1904) from southwestern China; and *N.chapaensis* (Bourret, 1937) and *N.lini* (Chou, 1999) from the northeastern Indochinese peninsula.

During our herpetological field surveys in Mt Dayao (MDY), Guangxi, south China, we collected a series of specimens of a small-sized frog that could be assigned to the genus *Nidirana* by possessing large suprabrachial gland in breeding males. Further detailed comprehensive analyses of molecules, bioacoustics, and morphology indicated that this frog was distinctive from all known congeners of *Nidirana*. Therefore, we propose it as a new species based on this study.

## Materials and methods

### Taxon sampling

**Figure 1.**
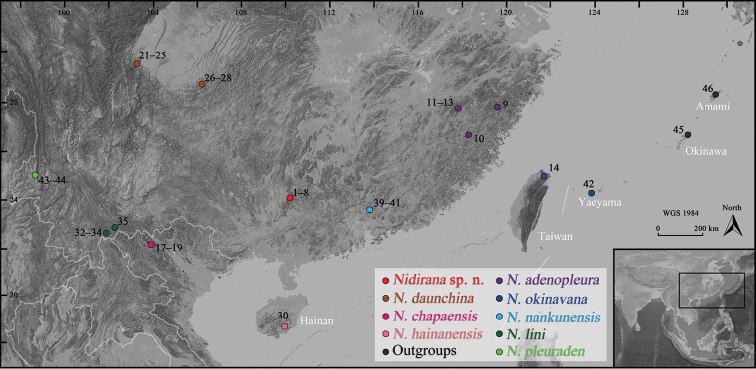
Localities of the samples used in this study. The numbers correspond to the ID numbers in Table 1.

Eight muscular samples of the unnamed species from MDY were used for molecular analysis. All samples were attained from euthanasia specimens and then preserved in 95% ethanol and stored at -40 °C. In addition, 36 sequences from all known *Nidirana* species and two sequences from the out-group *Babina* were obtained from GenBank and incorporated into our dataset. Detail information of these materials is shown in Table 1 and Fig. 1.

**Table 1.** Localities, voucher information, and GenBank numbers for all samples used in this study.

ID	Species	Localities (* = type localities)	Voucher	16S	CO1
**1**	*Nidiranayaoica* sp. nov.	China: Guangxi: Mt Dayao *	SYS a007009	MK882271	MK895036
**2**	*Nidiranayaoica* sp. nov.	China: Guangxi: Mt Dayao *	SYS a007011	MK882272	MK895037
**3**	*Nidiranayaoica* sp. nov.	China: Guangxi: Mt Dayao *	SYS a007012	MK882273	MK895038
**4**	*Nidiranayaoica* sp. nov.	China: Guangxi: Mt Dayao *	SYS a007013	MK882274	MK895039
**5**	*Nidiranayaoica* sp. nov.	China: Guangxi: Mt Dayao *	SYS a007014/CIB 110013	MK882275	MK895040
**6**	*Nidiranayaoica* sp. nov.	China: Guangxi: Mt Dayao *	SYS a007020	MK882276	MK895041
**7**	*Nidiranayaoica* sp. nov.	China: Guangxi: Mt Dayao *	SYS a007021	MK882277	MK895042
**8**	*Nidiranayaoica* sp. nov.	China: Guangxi: Mt Dayao *	SYS a007022	MK882278	MK895043
**9**	* Nidirana adenopleura *	China: Zhejiang: Jingning	SYS a002725	MF807827	MF807866
**10**	* Nidirana adenopleura *	China: Fujian: Nanping	SYS a005911	MF807844	MF807883
**11**	* Nidirana adenopleura *	China: Fujian: Mt Wuyi	SYS a005940	MF807851	MF807890
**12**	* Nidirana adenopleura *	China: Fujian: Mt Wuyi	SYS a005941	MF807852	MF807891
**13**	* Nidirana adenopleura *	China: Fujian: Mt Wuyi	XM2827	KF771281	/
**14**	* Nidirana adenopleura *	China: Taiwan: New Taipei	UMMZ 189963	DQ283117	/
**15**	* Nidirana adenopleura *	Not given	NMNS 2384	AF458118	/
**16**	* Nidirana adenopleura *	Not given	A-A-WZ001	NC018771	NC018771
**17**	* Nidirana chapaensis *	Vietnam: Lao Cai: Sapa *	ROM 28070	AF206460	/
**18**	* Nidirana chapaensis *	Vietnam: Lao Cai: Sapa *	1999.5871	KR827710	/
**19**	* Nidirana chapaensis *	Vietnam: Lao Cai: Sapa *	T2483/2000.4850	KR827711	KR087625
**20**	* Nidirana chapaensis *	Vietnam: Gia Lai	AMSR176027	KU840598	/
**21**	* Nidirana daunchina *	China: Sichuan: Mt Emei *	0609	KU840597	/
**22**	* Nidirana daunchina *	China: Sichuan: Mt Emei *	CIB-WU37990	DQ359988	/
**23**	* Nidirana daunchina *	China: Sichuan: Mt Emei *	HNNU 20060103	KF185065	/
**2**4	* Nidirana daunchina *	China: Sichuan: Mt Emei *	SYS a004594	MF807822	MF807861
**25**	* Nidirana daunchina *	China: Sichuan: Mt Emei *	SYS a004595	MF807823	MF807862
**2**6	* Nidirana daunchina *	China: Sichuan: Hejiang	SYS a004930	MF807824	MF807863
**27**	* Nidirana daunchina *	China: Sichuan: Hejiang	SYS a004931	MF807825	MF807864
**28**	* Nidirana daunchina *	China: Sichuan: Hejiang	SYS a004932	MF807826	MF807865
**29**	* Nidirana daunchina *	Not given	Not given	/	HQ395353
**30**	* Nidirana hainanensis *	China: Hainan: Mt Diaoluo *	SYS a003741	MF807821	MF807860
**31**	* Nidirana hainanensis *	China: Hainan	Not given	KU840596	/
**32**	* Nidirana lini *	China: Yunnan: Jiangcheng *	SYS a003967	MF807818	MF807857
**33**	* Nidirana lini *	China: Yunnan: Jiangcheng *	SYS a003968	MF807819	MF807858
**34**	* Nidirana lini *	China: Yunnan: Jiangcheng *	SYS a003969	MF807820	MF807859
**35**	* Nidirana lini *	China: Yunnan: Lyuchun	HNNULC001	KF185066	/
**36**	* Nidirana lini *	Laos: Xieng Khouang	FMNH256531	KR264073	/
**37**	* Nidirana lini *	Laos: Xieng Khouang	FMNH256532	KR264074	/
**38**	* Nidirana lini *	Not given	Not given	/	HQ395352
**39**	* Nidirana nankunensis *	China: Guangdong: Mt Nankun *	SYS a005717	MF807838	MF807877
**40**	* Nidirana nankunensis *	China: Guangdong: Mt Nankun *	SYS a005718	MF807839	MF807878
**41**	* Nidirana nankunensis *	China: Guangdong: Mt Nankun *	SYS a005719	MF807840	MF807879
**42**	* Nidirana okinavana *	Japan: Okinawa: Iriomote Island *	Not given	NC022872	NC022872
**43**	* Nidirana pleuraden *	China: Yunnan: Mt Gaoligong	SYS a003775	MF807816	MF807855
**44**	* Nidirana pleuraden *	China: Yunnan: Mt Gaoligong	SYS a003776	MF807817	MF807856
**45**	* Babina holsti *	Japan: Okinawa *	Not given	NC022870	NC022870
**46**	* Babina subaspera *	Japan: Kagoshima: Amami Island *	Not given	NC022871	NC022871

### DNA Extraction, PCR amplification, and sequencing

Genomic DNA were extracted from muscle tissue samples, using DNA extraction kit from Tiangen Biotech (Beijing) Co., Ltd. Two mitochondrion genes, namely partial 16S ribosomal RNA gene (16S) and partial cytochrome C oxidase 1 gene (CO1), were amplified. Primers used for 16S were L3975 (5’-CGCCTGTTTACCAAAAACAT-3’) and H4551 (5’-CCGGTCTGAACTCAGATCACGT-3’), and L2A (5’-CCAAACGAGCCTAGTGATAGCTGGTT-3’) and H10 (5’-TGATTACGCTACCTTTGCACGGT-3’), and for CO1 were Chmf4 (5’-TYTCWACWAAYCAYAAAGAYATCGG-3’) and Chmr4 (5’-ACYTCRGGRTGRCCRAARAATCA-3’), and dgLCO (5’-GGTCAACAAATCATAAAGAYATYGG-3’) and dgHCO (5’-AAACTTCAGGGTGACCAAARAAYCA-3’), following Lyu et al. (2019). PCR amplifications were processed with the cycling conditions that initial denaturing step at 95 °C for 4 min, 35 cycles of denaturing at 94 °C for 40 s, annealing at 53 °C (for 16S) / 48 °C (for CO1) for 40 s and extending at 72 °C for 60 s, and a final extending step at 72 °C for 10 min. PCR products were purified with spin columns and then sequenced with both forward and reverse primers using BigDye Terminator Cycle Sequencing Kit per the guidelines, on an ABI Prism 3730 automated DNA sequencer by Shanghai Majorbio Bio-pharm Technology Co, Ltd. All sequences were deposited in GenBank (Table 1).

### Phylogenetic analyses

DNA sequences were aligned by the Clustal W algorithm with default parameters (Thompson et al. 1997) and trimmed with the gaps partially deleted in MEGA 6 (Tamura et al. 2013). Two gene segments, 1041 base pairs (bp) of 16S and 573 bp of CO1, were concatenated seriatim into a 1614-bp sequence, and further divided into four partitions by codons. The partitions were tested in jmodeltest v2.1.2 with Akaike and Bayesian information criteria, all resulting the best-fitting nucleotide substitution models of GTR+I+G. Sequenced data was analyzed using Bayesian inference (BI) in MrBayes 3.2.4 (Ronquist et al. 2012), and maximum likelihood (ML) in RaxmlGUI 1.3 (Silvestro and Michalak 2012). Two independent runs were conducted in a BI analysis, each of which was performed for 10,000,000 generations and sampled every 1000 generations with the first 25% samples were discarded as burn-in, resulting a potential scale reduction factor (PSRF) of < 0.005. In ML analysis, the bootstrap consensus tree inferred from 1000 replicates was used to represent the evolutionary history of the taxa analyzed.

### Bioacoustic analysis

Advertisement calls of the specimen SYS a007009 from MDY were recorded in the field at the air temperature of 18 °C using a SONY PCM D100 digital sound recorder. The sound files in wave format were sampled at 44.1 kHz with 24 bits in depth. Praat 6.0.27 (Boersma 2001) was used to obtain the oscillogram, sonogram, and power spectrum (window length = 0.005 s). Raven pro 1.5 (Cornell Lab of Ornithology, 2003–2014) was used to quantify the acoustic properties (window size = 256 points, fast Fourier transform, Hanning window with no overlap). The following measurements were taken for each call: call duration (the time between onset of the first note and offset of the last note in a call) and call PF (peak frequency; the frequency at which max power occurs within the call); the following measurements were taken for each note: note duration (the time between onset and offset of a note), note rise time (the time between onset and max amplitude of a note), note interval (the time between adjacent notes in a call), note PF and note IQR-BW (inter-quartile range bandwidth; the difference between the first and third quartile frequencies within a note). Mean and standard deviation (SD) were calculated in R 3.3.2 (R Core Team 2016).

### Morphology

Comparison characters of all known congeners were obtained from the literature (Boettger 1895; Boulenger 1904, 1909; Schmidt 1925; Chang and Hsu 1932; Bourret 1937; Kuramoto 1985; Chou 1999; Fei et al. 2007, 2009; Matsui 2007; Chuaynkern et al. 2010; Lyu et al. 2017) and 55 examined museum specimens of six species which are listed in the Appendix 1. All specimens were fixed in 10% buffered formalin and later transferred to 70% ethanol, and deposited in the Museum of Biology, Sun Yat-sen University (**SYS**), Natural History Museum of Guangxi (**NHMG**), and Chengdu Institute of Biology, Chinese Academy of Sciences (**CIB**), China.

Morphological descriptions follow the consistent definition by Fei et al. (2009), Chuaynkern et al. (2010) and Lyu et al. (2017). External measurements were made for the unnamed specimens with digital calipers (Neiko 01407A Stainless Steel 6-Inch Digital Caliper, USA) to the nearest 0.1 mm. Mean and standard deviation (SD) were calculated in R 3.3.2 (R Core Team 2016). These measurements were as follows:

**SVL** snout-vent length (from tip of snout to posterior margin of vent);

**HDL** head length (from tip of snout to the articulation of the jaw);

**HDW** head width (head width at the commissure of the jaws);

**SNT** snout length (from tip of snout to the anterior corner of the eye);

**IND** internasal distance (distance between nares);

**IOD** interorbital distance (minimum distance between upper eyelids);

**ED** eye diameter (from the anterior corner of the eye to posterior corner of the eye);

**TD** tympanum diameter (horizontal diameter of tympanum);

**TED** tympanum-eye distance (from anterior edge of tympanum to posterior corner of the eye);

**HND** hand length (from the proximal border of the outer palmar tubercle to the tip of digit III);

**RAD** radio-ulna length (from the flexed elbow to the proximal border of the outer palmar tubercle);

**FTL** foot length (from distal end of shank to the tip of digit IV);

**TIB** tibial length (from the outer surface of the flexed knee to the heel).

Sex and age were determined by secondary sexual characters, i.e., the presence of suprabrachial glands in males. Webbing formula was written according to Savage (1975).

## Results

**Figure 2.**
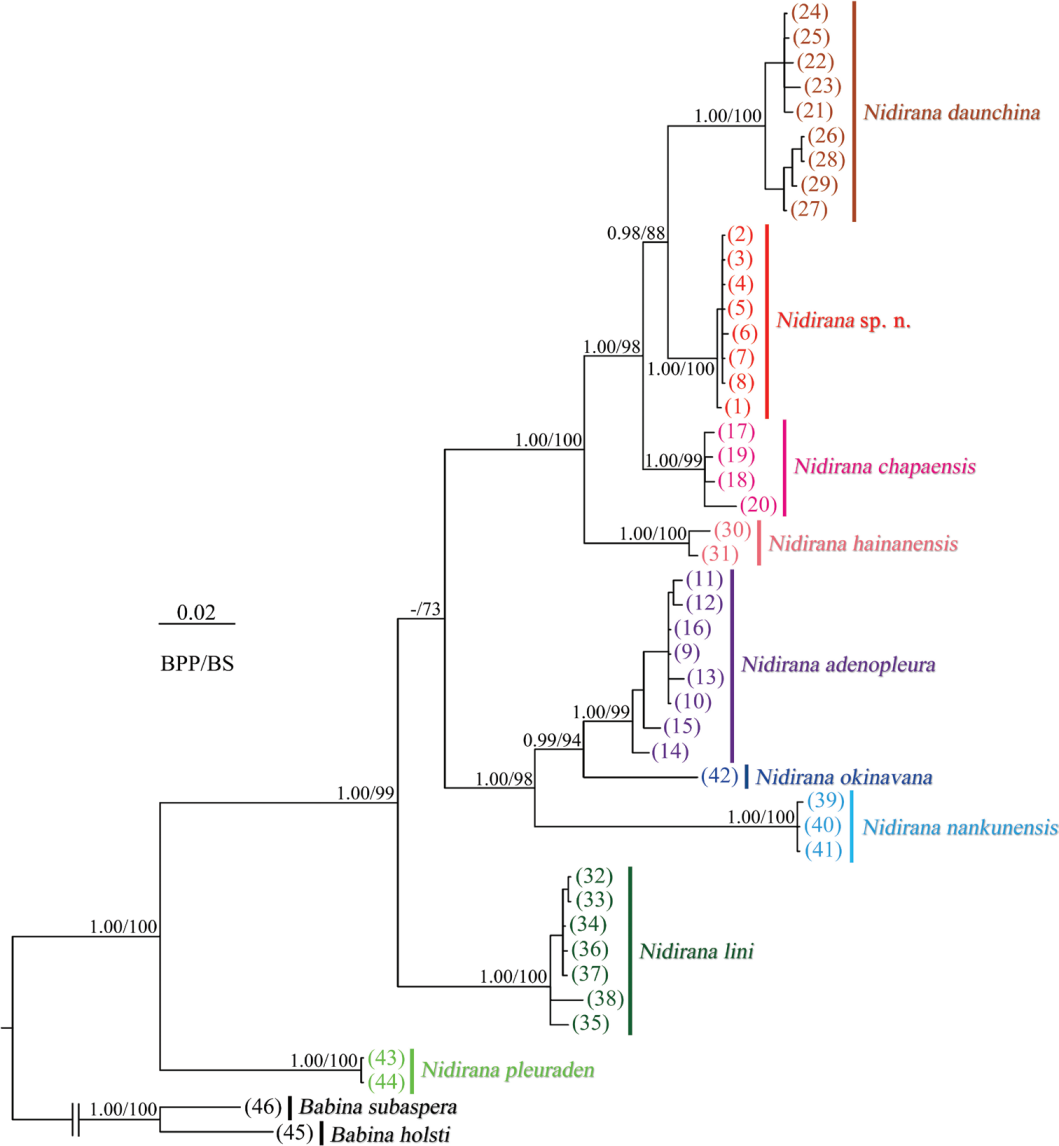
Bayesian inference and maximum-likelihood phylogenies. Number in parenthesis corresponds to the ID number in Table 1.

The ML and BI analyses resulted in essentially identical topologies and were integrated in Fig. 2, in which the major nodes were sufficiently supported with the Bayesian posterior probabilities (BPP) > 0.95 and the bootstrap supports (BS) for maximum likelihood analysis > 70. This mitochondrial result is consistent with the phylogenic relationship in Lyu et al. (2017). The *Nidirana* specimens from MDY, southern China, grouped in a clade with strong supported values and small divergences, forming a sister taxon to *N.daunchina* from western China, then together forming the sister taxon to *N.chapaensis* from northern Vietnam.

Morphologically, the specimens from MDY significantly differ from the recognized congeners by the following characteristics: (1) medium-sized body, SVL 40.4–45.9 mm in adult males vs. 33.3–37.1 mm in *N.nankunensis*; (2) finger IV longer than finger I vs. equal in *N.chapaensis*; (3) presence of lateroventral groove on every digit vs. absent on fingers and toes in *N.pleuraden*; absent or barely visible on fingers in *N.daunchina*; absent on finger I in *N.chapaensis*, *N.lini*, *N.nankunensis*, *N.adenopleura*, and *N.okinavana*; (4) tibio-tarsal articulation reaches the nostril vs. beyond the snout tip in *N.lini*; (5) the presence of a single nuptial pad vs. absent in *N.hainanensis*; divided into two parts in *N.chapaensis*; (6) the presence of a pair of subgular vocal sacs vs. absent in *N.okinavana*; (7) the absence of spinules on dorsal skin vs. present in *N.adenopleura*, *N.lini* and *N.pleuraden*. Detail comparison between the specimens from MDY and its congeners is listed in Table 2 with the characteristics item by item.

**Table 2.** Diagnostic characters separating *Nidiranayaoica* sp. nov. from congeners.

Characteristics	* N. yaoica *	* N. daunchina *	* N. chapaensis *	* N. hainanensis *	* N. adenopleura *	* N. nankunensis *	* N. okinavana *	* N. lini *	* N. pleuraden *
SVL of males	40.4–45.9	40.6–51.0	35.5–42.5	32.8–44.4	43.1–57.6	33.3–37.1	35.5–42.8	44.1–63.1	45.4–58.7
SVL of females	/	44.0–53.0	41.0–51.8	?	47.6–60.7	37.8–39.5	44.6–48.8	57.7–68.6	45.5–62.5
Body habitus	Stocky	Stocky	Stocky	Stocky	Elongated	Stocky	Stocky	Elongated	Elongated
Fingers tips	Dilated	Dilated	Dilated	Dilated	Dilated	Dilated	Dilated	Dilated	Not dilated
Lateroventral groove on fingers	Present	Absent or rarely present	Present except finger I	Present	Present except finger I	Present except finger I	Present except finger I	Present except finger I	Absent
Relative length of fingers	II < I < IV < III	II < I < IV < III	II < I = IV < III	II < I < IV < III	II < I < IV < III	II < I < IV < III	II < I < IV < III	II < I < IV < III	II < I < IV < III
Toes tips	Dilated	Dilated	Dilated	Dilated	Dilated	Dilated	Dilated	Dilated	Not dilated
Lateroventral groove on toes	Present	Present	Present	Present	Present	Present	Present	Present	Absent
Relative length of toes	I < II < V < III < IV	I < II < V < III < IV	I < II < V < III < IV	I < II < V < III < IV	I < II < V < III < IV	I < II < V < III < IV	I < II < V < III < IV	I < II < V < III < IV	I < II < V < III < IV
Tibio-tarsal articulation	Nostril	Nostril	Nostril	Nostril	Snout tip or eye-snout	Nostril	Eye center-near nostril	Beyond snout	Eye-snout
Subgular vocal sacs	Present	Present	Present	Present	Present	Present	Absent	Present	Present
Nuptial pad	One	One	Two	Absent	One	One	Poorly one	One	One
Spinules on dorsal skin	Absent	Absent	Absent or few above vent	Absent	Entire or posterior dorsal skin	Absent or few above vent	Absent	Posterior dorsal skin	Posterior dorsal skin
Nest construction	?	Present	Present	Present	Absent	Present	Present	Absent	Absent
Tadpole labial tooth row formula	?	1:1+1/1+1:2 or 1:1+1/2+2:1	1:1+2/1+1:2	?	1:1+1/1+1:2 or 1:0+0/1+1:1	1:1+1/1+1:2	1:1+1/1+1:2	1:1+1/1+1:2	1:1+1/1+1:2 or 1:1+1/2+2:1
Calling	1–3 fast-repeated notes	2–5 notes containing a specific first note	3 notes	2–4 fast-repeated double-notes	2–4 notes	13–15 fast-repeated notes containing a specific first note	10–25 fast-repeated notes	5–7 notes	4–7 notes
Cites	This study	Liu (1950); Fei et al. (2009); Lyu et al. (2017)	Chuaynkern et al. (2010)	Fei et al. (2009, 2012); Lyu et al. (2017)	Pope (1931); Chuaynkern et al. (2010); Lyu et al. (2017)	Lyu et al. (2017)	Matsui and Utsunomiya (1983); Chuaynkern et al. (2010); Lyu et al. (2017)	Chou (1999); Fei et al. (2009); Lyu et al. (2017)	Fei et al. (2009); Lyu et al. (2017)

Further, the advertisement call from the frogs from MDY is different from the congeners by: (1) containing 1–3 fast-repeated identical regular notes (vs. containing 2–4 fast-repeated double-notes in *N.hainanensis*; containing a significantly different first note in *N.daunchina* and *N.nankunensis*); (2) the call notes last 30–54 ms vs. call notes last 115–252 ms in *N.adenopleura*; the first notes last 108–135 ms in *N.nankunensis*; the first notes last 162–197 ms and the others last 131–150 ms in *N.daunchina*; (3) the intervals between notes last 212–372 ms vs. last 98–213 ms in *N.adenopleura*; last 12–166 ms in *N.nankunensis*.

Therefore, based on the molecular, morphological, and bioacoustic differences, the specimens from MDY, southern China, represent an unnamed species which is described as a new species of genus *Nidirana*.

### Taxonomy account

#### Nidirana yaoica sp. nov.

Taxon classificationAnimaliaAnuraRanidae

http://zoobank.org/D05423B2-1812-4AF4-890C-A0A1915BD8A6

##### Chresonymy.

*Nidiranaadenopleura*: Fei et al. 2009 (Mt. Dayao, Jinxiu, Guangxi); Mo et al. 2014 (Jinxiu, Guangxi)

##### Holotype.

**Figure 3.**
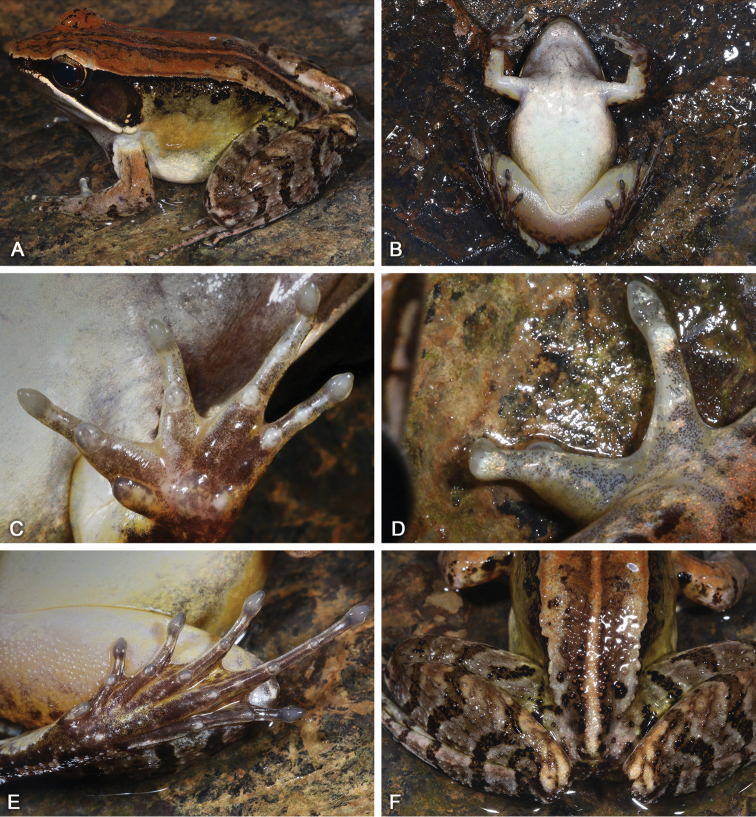
Morphological features of the adult male holotype SYS a007022 of *Nidiranayaoica* sp. nov. in life. **A** dorsolateral view **B** ventral view **C** left hand **D** poorly developed nuptial pad **E** left foot **F** surface of posterior dorsum and hind limbs.

SYS a007022 (Fig. 3), adult male, collected by Zhi-Tong Lyu on 1 June 2018 from Mt Dayao (24.1602N, 110.2304E; ca 1190 m a.s.l.), Jinxiu Yao Autonomous County, Guangxi Zhuang Autonomous Region, China.

##### Paratypes.

SYS a007009, 7011–7013, 7020–7021, SYS a007014/CIB 110013, seven adult males collected by Zhi-Tong Lyu, Yu-Long Li and Cheng-Yu Yang on 30 May–1 June 2018 from the same locality as the holotype. NHMG 1503043–47, five adult males collected by Yun-Ming Mo and Wei-Cai Chen on 19 March 2015 from the neighboring locality as the holotype (24.1035N,110.2294E; ca 1350 m a.s.l.).

##### Etymology.

The specific name *yaoica* is an adjective derived from Yao, referring to the type locality of the new species, Mt Dayao in Jinxiu Yao Autonomous County, where the settlement of the Yao people is located. We suggest its English common name to be Mt Dayao music frog and its Chinese name Yao Qin Wa (瑶琴蛙).

##### Differential diagnosis.

*Nidiranayaoica* sp. nov. is distinguished from its congeners by the following combination of the morphological characteristics: (1) body medium-size and stocky, with SVL 43.8 ± 1.7 (40.4–45.9, n = 13) mm in adult males; (2) disks of digits dilated, pointed; (3) lateroventral grooves present on every digit; (4) heels overlapping; (5) tibio-tarsal articulation reaching at the nostril; (6) mid-dorsal stripe present; (7) posterior of dorsal skin rough with dense tubercles but without spinules; (8) week supernumerary tubercles below the base of fingers III and IV, palmar tubercles prominent and distinct; (9) a pair of subgular vocal sacs present; (10) one single nuptial pad present on the first finger, nuptial spinules invisible; (11) suprabrachial gland large; (12) calling: 1–3 fast-repeated regular notes.

##### Description of holotype.

Adult male. Body stocky, SVL 44.6 mm; head longer than wide (HDW/HDL 0.92), flat above; snout rounded in dorsal and lateral views, slightly protruding beyond lower jaw, longer than horizontal diameter of eye (SNT/ED 1.26); canthus rostralis distinct, loreal region concave; nostril round, directed laterally, closer to the snout than to the eye; a longitudinal swollen mandibular ridge extending from below nostril through lower edges, eye and tympanum to above insertion of arm, where the ridge is intermittent, forming a maxillary gland and shoulder gland; supratympanic fold absent; interorbital space flat, narrower than internasal distance (IND/IOD 1.37); pupil elliptical, horizontal; tympanum distinct, round, TD/ED 0.72, and close to eye, TED/TD 0.38; pineal ocellus present; vomerine ridge present, bearing small teeth; tongue large, cordiform, notched behind.

Forelimbs moderately robust, lower arm 19% of SVL and hand 27% of SVL; fingers thin, relative finger lengths II < I < IV < III; tip of each finger slightly dilated and remarkable elongated, forming long pointed disks; well-developed lateroventral grooves on all fingers, not meeting at the tip of disks; fingers free of webbing; presence of weak lateral fringes on inner and outer sides of fingers II, III and IV, and on outer side of finger I; subarticular tubercles prominent and rounded; week supernumerary tubercles below the base of fingers III and IV; three elliptic, large, prominent and very distinct palmar tubercles.

Hindlimbs relatively robust, tibia 53% of SVL and foot 78% of SVL; heels overlapping when hindlimbs flexed at right angles to axis of body; tibio-tarsal articulation reaching the nostril when hindlimb is stretched along the side of the body; toes relatively long and thin, relative lengths I < II < V < III < IV; tip of each toe slightly dilated with remarkable elongated ventral callous pad, forming long and pointed disk; well-developed lateroventral grooves on toes , not meeting at the tip of disks; webbing moderate, webbing formula: I 2 - 2½ II 1⅔ - 3 III 2⅓ - 3½ IV 3½ - 2 V; presence of lateral fringes on inner and outer sides of each toes, forming distinct dermal flap on the lateral edges of toes I and V; subarticular tubercles rounded, prominent; inner metatarsal tubercle elliptic, twice as long its width; outer metatarsal tubercle indistinct, small and rounded; tarsal folds and tarsal tubercle absent.

Dorsal skin of head and anterior body smooth, posterior dorsum of body rough with dense tubercles but not bearing horny spinules; developed dorsolateral fold from posterior margin of upper eyelid to above groin but intermittent posteriorly; flank relatively smooth with dense tubercles on region nearly the dorsolateral fold; a large and smooth suprabrachial gland behind base of forelimb, slightly prominent; dorsal surface of upper arm with two longitudinal ridges and slightly extending to lower arm; the dorsal surfaces of thigh and tibia with several longitudinal ridges and tubercles bearing spinules. Ventral surface of head, body, and limbs smooth; large flattened tubercles densely arranged on the rear of thigh and around vent.

##### Color in life of holotype.

Dorsal surface of head and body reddish brown; pineal ocellus yellowish; a longitudinal reddish brown mid-dorsal stripe edged with broad dark brown, beginning from snout, across pineal ocellus, posteriorly extending to vent; several black spots on upper eyelids and posterior dorsum of body; dorsolateral fold bicolor, upper part reddish brown and lower part black; upper flank yellowish brown with irregular black spots; lower flank yellowish white; suprabrachial gland yellowish brown. Dorsal forelimbs reddish brown; a longitudinal black stripe on the anterior surface of the forelimb; irregular black marks on dorsal surface of the forelimb; dorsal hindlimbs non-uniform dark brown, four black crossbars on the thigh, three on the tibia and three on the tarsus; irregular black marks on dorsal toes. Loreal and temporal regions black, tympanum dark brown; upper ⅓ iris bright brownish white and lower ⅔ iris reddish brown; maxillary gland and shoulder gland yellowish white. Lips and throat grey white, but two subgular vocal sacs slightly dark colored; ventral surface of body and limbs creamy white; rear thigh tinged with pink; ventral hand and foot pale white with large black patches.

##### Color in preservative of holotype.

Dorsal surface faded, but dark brown edges of the mid-dorsal stripe more distinct; black spots on dorsum more distinct; upper flank black; limbs faded, the crossbars clearer; ventral surface faded, throat and posterior of chest with smoky gray markings.

##### Variations.

**Figure 4.**
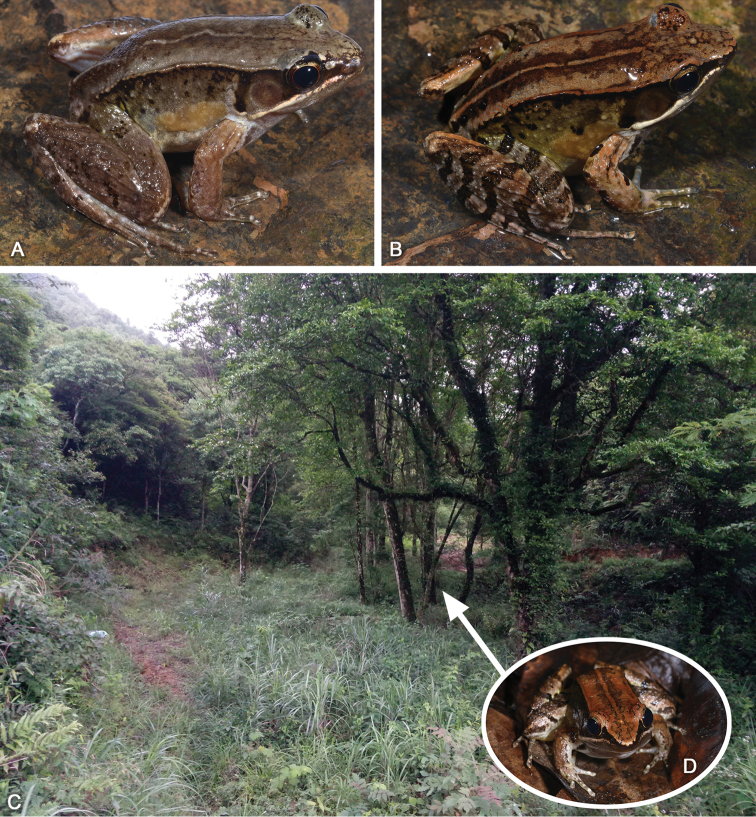
**A, B** paratypes SYS a007009 and SYS a007021 of *Nidiranayaoica* sp. nov. **C** habitat of *Nidiranayaoica* sp. nov. in the type locality in Mt Dayao **D** the holotype SYS a007022 in wild.

Measurements of type series are given in Table 3. All specimens were similar in morphology. Dorsal surface light brown in SYS a007009 (Fig. 4A), 7011, 7013 and 7020; mid-dorsal stripe begins from pineal ocellus in SYS a007011, 7013, 7014, 7020 and 7021 (Fig. 4B), unclear in SYS a007009; pineal ocellus invisible in SYS a007009.

**Table 3.** Measurements (in mm) of the type series of *Nidiranayaoica* sp. nov.

Specimens No.	SYS a007022 (holotype)	SYS a007009	SYS a007011	SYS a007012	SYS a007013	SYS a007014 /CIB 110013	SYS a007020	SYS a007021	NHMG 1503043	NHMG 1503044	NHMG 1503045	NHMG 1503046	NHMG 1503047	summarizing of measurement (minimum-maximum, mean ± SD)
**Sex**	Male	Male	Male	Male	Male	Male	Male	Male	Male	Male	Male	Male	Male	Males
**SVL**	44.6	42.1	44.1	44.9	43.2	45.5	45.6	44.6	42.6	40.4	43.9	42.5	45.9	40.4–45.9, 43.8 ± 1.7
**HDL**	17.6	16.4	18.2	16.8	16.3	18.6	17.5	16.9	16.2	15.7	16.2	16.3	17.7	15.7–18.6, 16.9 ± 0.9
**HDW**	16.2	15.3	16.4	16.2	15.0	16.7	16.3	16.1	16.0	15.6	15.7	15.7	17.2	15.0–17.2, 16.0 ± 0.6
**SNT**	6.8	6.2	6.8	6.7	6.9	7.2	6.2	7.0	7.5	7.7	7.7	7.7	8.7	6.2–8.7, 7.2 ± 0.7
**IND**	5.6	5.9	5.8	6.0	5.8	5.4	5.5	5.6	6.0	6.2	6.3	6.0	6.6	5.4–6.6, 5.9 ± 0.3
**IOD**	4.1	4.3	4.9	4.7	5.1	4.8	4.4	4.5	4.2	3.8	3.5	3.5	4.2	3.5–5.1, 4.3 ± 0.5
**ED**	5.4	5.1	5.2	4.7	4.6	5.4	5.4	5.0	5.3	5.1	5.2	5.1	5.0	4.6–5.4, 5.1 ± 0.2
**TD**	3.9	3.2	3.4	3.7	3.9	3.9	3.6	3.9	4.1	4.1	4.2	4.1	4.5	3.2–4.5, 3.9 ± 0.4
**TED**	1.5	1.3	1.0	1.3	1.2	1.2	1.6	1.2	1.1	1.1	1.1	1.2	1.6	1.0–1.6, 1.2 ± 0.2
**HND**	12.0	10.3	11.1	10.4	10.3	11.5	10.9	12.4	10.4	10.9	12.0	10.2	12.8	10.2–12.8, 11.1 ± 0.9
**RAD**	8.4	8.8	9.4	8.6	8.4	8.6	8.6	8.7	8.2	8.0	7.8	8.3	8.4	7.8–9.4, 8.5 ± 0.4
**FTL**	34.9	33.1	34.3	34.6	34.3	35.0	33.7	35.7	32.3	3.1	32.9	32.3	35.6	3.1–35.7, 31.4 ± 9.0
**TIB**	23.6	23.5	23.0	22.6	23.2	23.9	23.5	23.4	22.0	21.6	22.9	22.4	25.6	21.6–25.6, 23.1 ± 1.0
**HDL/SVL**	0.39	0.39	0.41	0.37	0.38	0.41	0.38	0.38	0.38	0.39	0.37	0.38	0.39	0.37–0.41, 0.39 ± 0.01
**HDW/SVL**	0.36	0.36	0.37	0.36	0.35	0.37	0.36	0.36	0.38	0.39	0.36	0.37	0.37	0.35–0.39, 0.37 ± 0.01
**HDW/HDL**	0.92	0.94	0.90	0.96	0.92	0.89	0.93	0.95	0.99	0.99	0.97	0.96	0.97	0.89–0.99, 0.95 ± 0.03
**SNT/HDL**	0.39	0.38	0.38	0.40	0.42	0.39	0.35	0.41	0.46	0.49	0.48	0.47	0.49	0.35–0.49, 0.43 ± 0.05
**SNT/SVL**	0.15	0.15	0.15	0.15	0.16	0.16	0.14	0.16	0.18	0.19	0.18	0.18	0.19	0.14–0.19, 0.16 ± 0.02
**IND/HDW**	0.35	0.38	0.36	0.37	0.38	0.32	0.34	0.35	0.38	0.40	0.40	0.38	0.38	0.32–0.40, 0.37 ± 0.02
**IOD/HDW**	0.25	0.28	0.30	0.29	0.34	0.29	0.27	0.28	0.26	0.24	0.22	0.22	0.24	0.22–0.34, 0.27 ± 0.03
**ED/HDL**	0.31	0.31	0.28	0.28	0.28	0.29	0.31	0.30	0.33	0.32	0.32	0.31	0.28	0.28–0.33, 0.30 ± 0.02
**ED/SVL**	0.12	0.12	0.12	0.10	0.11	0.12	0.12	0.11	0.12	0.13	0.12	0.12	0.11	0.10–0.13, 0.12 ± 0.01
**TD/ED**	0.72	0.62	0.66	0.79	0.85	0.73	0.67	0.78	0.77	0.80	0.81	0.80	0.90	0.62–0.90, 0.77 ± 0.08
**TED/TD**	0.38	0.42	0.29	0.35	0.31	0.29	0.43	0.31	0.27	0.27	0.26	0.29	0.36	0.26–0.43, 0.32 ± 0.06
**HND/SVL**	0.27	0.24	0.25	0.23	0.24	0.25	0.24	0.28	0.24	0.27	0.27	0.24	0.28	0.23–0.28, 0.25 ± 0.02
**RAD/SVL**	0.19	0.21	0.21	0.19	0.19	0.19	0.19	0.20	0.19	0.20	0.18	0.20	0.18	0.18–0.21, 0.19 ± 0.01
**FTL/SVL**	0.78	0.79	0.78	0.77	0.79	0.77	0.74	0.80	0.76	0.08	0.75	0.76	0.78	0.08–0.80, 0.71 ± 0.20
**TIB/SVL**	0.53	0.56	0.52	0.50	0.54	0.52	0.52	0.52	0.52	0.53	0.52	0.53	0.56	0.50–0.56, 0.53 ± 0.02

##### Male secondary sexual characteristics.

A pair of subgular vocal sacs, a pair of slit-like openings at posterior of jaw; a single light brown nuptial pad on the dorsal surface of first finger, nuptial spinules invisible; suprabrachial gland present.

##### Distribution and ecology.

Currently, *Nidiranayaoica* sp. nov. is known only from the type locality, Mt Dayao, Jinxiu, Guangxi, in southern China. This frog inhabits in the swamps and ponds surrounded by moist subtropical secondary evergreen broadleaved forests (Fig. 4C, D). The adult male calls in the brushwood at the bank, from mid-March to late May. Nevertheless, the females, tadpoles, and much of the ecology and behavior of this species remain unknown.

##### Vocalization.

**Figure 5.**
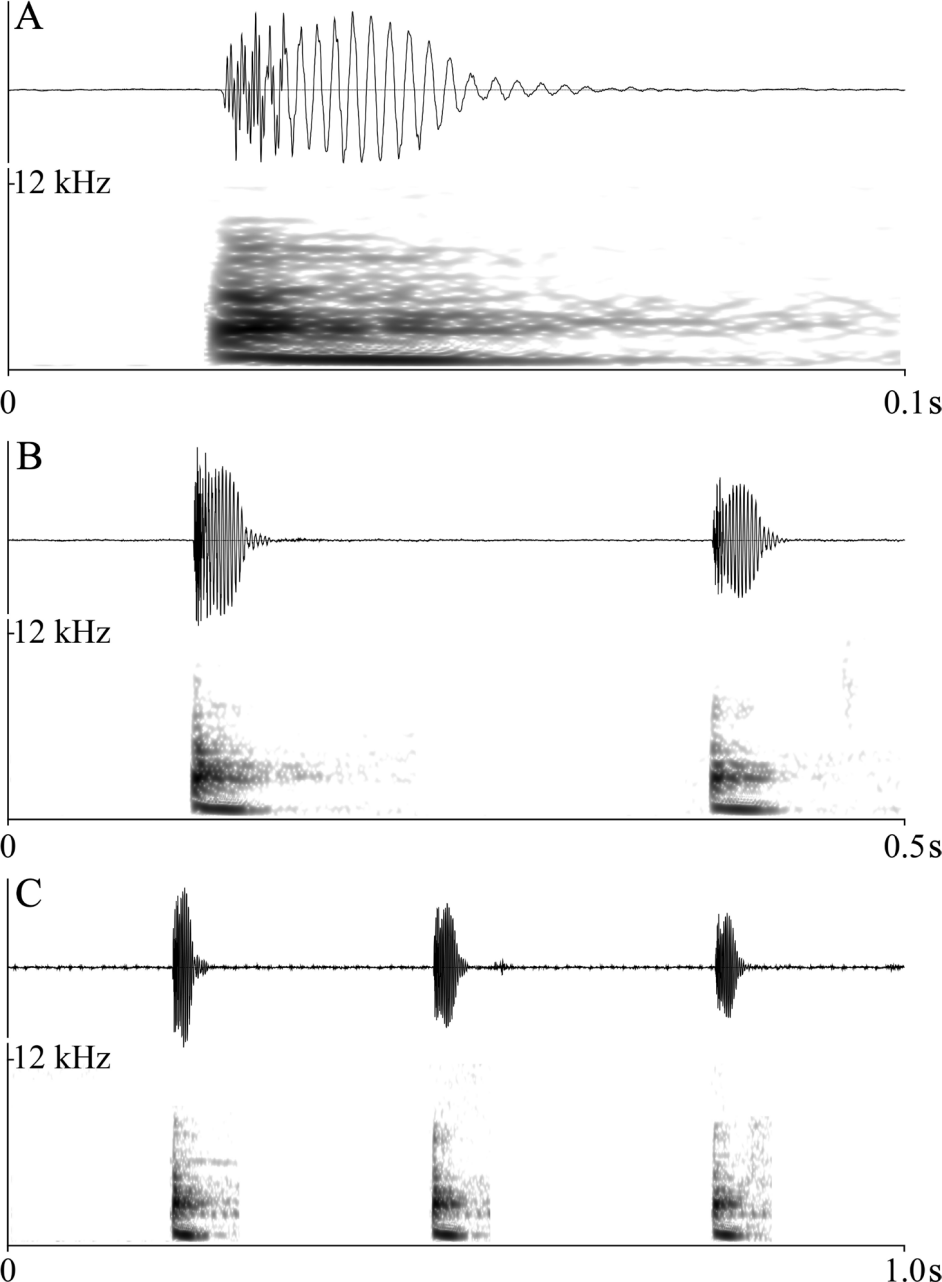
Advertisement call spectrograms of *Nidiranayaoica* sp. nov. **A** one-note call **B** two-note call; **C** three-note call.

The call spectrograms are shown in Fig. 5 and the measurement parameters are listed in Table 4. The advertisement call (n = 87) of *Nidiranayaoica* sp. nov. contains 1–3 rapidly repeated, identical, regular notes with the PF of 516.8 Hz and note IQR-BW of 172.3 Hz or 0 generally. The one-note call (n = 25) has a duration of 43.3 ± 2.7 ms with the rise time of 10.1 ± 4.5 ms. The two-note call (n = 59) has a duration of 355.9 ± 31.1 ms; the first note lasts 43.5 ± 2.8 ms with the rise time of 8.5 ± 4.6 ms, and the second lasts 39.6 ± 3.3 ms with the rise time of 11.6 ± 4.4 ms; the note interval last 272.8 ± 31.7 ms.

**Table 4.** Vocalization parameters of paratype SYS a007009 of *Nidiranayaoica* sp. nov.

	one-note call (n = 25)	two-note call (n = 59)	three-note call (n = 3)
**Call duration (ms)**	37–51, 43.3 ± 2.7	307–454, 355.9 ± 31.1	565–678, 628.0 ± 57.6
**Note duration (ms)**	37–51, 43.3 ± 2.7	1^st^ note: 36–51, 43.5 ± 2.8;	1^st^ note: 42–54, 46.7 ± 6.4;
		2^nd^ note: 30–49, 39.6 ± 3.3	2^nd^ note: 37–40, 38.7 ± 1.5;
			3^rd^ note: 35–52, 42.3 ± 8.7
**Note rise time (ms)**	1.6–15.5, 10.1 ± 4.5	1^st^ note: 2.0–16.0, 8.5 ± 4.6;	1^st^ note: 3.7–13.7, 7.4 ± 5.5;
		2^nd^ note: 1.7–17.9, 11.6 ± 4.4	2^nd^ note: 13.1–15.8, 14.8 ± 1.5;
			3^rd^ note: 14.0–16.1, 15.3 ± 1.1
**Note interval (ms)**	/	215–372, 272.8 ± 31.7	1^st^ interval: 212–250, 234.0 ± 19.7;
			2^nd^ interval: 222–302, 266.3 ± 40.7
**Call PF (Hz)**	516.8	516.8	516.8
**Note PF (Hz)**	516.8	1^st^ note: 516.8 (98.3%) or 2584 (1.7%);	1^st^ note: 516.8;
		2^nd^ note: 516.8	2^nd^ note: 516.8;
			3^rd^ note: 516.8
**Note IQR-BW (Hz)**	172.3 (48.0%) or 0 (52.0%)	1^st^ note: 344.5 (8.4%), 172.3 (45.8%) or 0 (45.8%);	1^st^ note: 172.3 (33.3%) or 0 (66.6%);
		2^nd^ note: 172.3 (54.2%) or 0 (45.8%)	2^nd^ note: 172.3 (33.3%) or 0 (66.6%);
			3^rd^ note: 172.3 (33.3%) or 0 (66.6%)

## Discussion

The taxonomic status for the *Nidirana* population in MDY was suspected and suggested a further study by Fei et al. (2009), despite their work reported it as *N.adenopleura* tentatively which was followed by Mo et al. (2014). Currently this population is revealed as *N.yaoica* sp. nov. in present work. In morphology, this frog is similar to *N.hainanensis* by the presence of lateroventral groove on all digits, and further to *N.daunchina*, *N.chapaensis*, and *N.okinavana* by the absence of spinules on dorsal skin. Bioacoustically, *N.yaoica* sp. nov. has the same calling pattern as *N.adenopleura*, which contains several fast-repeated identical regular notes, but different from the pattern in *N.daunchina* and *N.hainanensis*. The phylogenetic tree showed that the new species is closer to *N.daunchina* with moderate supports (BPP 0.98 and BS 88), and then to *N.chapaensis* and *N.hainanensis*.

The genus *Nidirana* was recognized as a distinct genus recently based on comprehensive evidence by Lyu et al. (2017). For the interspecific relationship within the genus, Dubois (1992) constructed two morphological species groups: *N.pleuraden* group for *N.pleuraden* and *N.adenopleura* group for the other known species; Fei et al. (2009) proposed *N.daunchina* group for *N.daunchina* and *N.psaltes* Kuramoto, 1985 (= *N.okinavana*), and *N.adenopleura* group for *N.adenopleura*, *N.lini*, and *N.hainanensis*, but excluding *N.chapaensis*, and placing *N.pleuraden* in another genus *Pelophylax* Fitzinger, 1843; Chuaynkern et al. (2010) suggested three species groups for Music frogs based on the morphological characters and ecological behavior of nest construction: *N.pleuraden* group for *N.pleuraden*, *N.adenopleura* group for *N.adenopleura* and *N.lini*, and *N.okinavana* group for *N.daunchina*, *N.okinavana*, and *N.chapaensis*. From the current mitochondrial results (Lyu et al. 2017; this study), the *N.pleuraden* consistently formed the basal lineage of this genus, while the monophyly of the three species groups *N.adenopleura* group (Fei et al. 2009; Chuaynkern et al. 2010), *N.okinavana* group, and *N.daunchina* group, was challenged. The main conflicts are: (1) *N.okinavana* was suggested morphologically more similar to *N.daunchina* and *N.chapaensis* (Fei et al. 2009; Chuaynkern et al. 2010) while clustered with *N.adenopleura* in the phylogeny; (2) *N.hainanensis* was suggested morphologically more similar to *N.adenopleura* and *N.lini* (Fei et al. 2009) while clustered with *N.daunchina* and *N.chapaensis* in the phylogeny; (3) *N.lini* was suggested morphologically more similar to *N.adenopleura* (Fei et al. 2009; Chuaynkern et al. 2010) while formed the basal lineage of the congeners except *N.pleuraden* in the phylogeny.

Thus we propose to follow Dubois’s (1992) suggestion, regarding two species groups within the genus *Nidirana*: (1) *N.pleuraden* group, the lateroventral groove absent on fingers and toes: one species, *N.pleuraden*; (2) *N.adenopleura* group, the lateroventral groove present on toes, absent or present on fingers: eight species, *N.adenopleura*, *N.okinavana*, *N.nankunensis*, *N.hainanensis*, *N.chapaensis*, *N.daunchina*, *N.yaoica* sp. nov., and *N.lini*.

## Supplementary Material

XML Treatment for Nidirana yaoica

## Appendix 1

**Specimens examined**

*Nidiranaadenopleura* (29): **China: Fujian**: Yanping District (type locality): SYS a005911–5916; Mt Wuyi: SYS a005939–5943; Jiangshi Nature Reserve: SYS a004112, 4132; Mt Yashu: SYS a005890–5891, 5901–5902; **Jiangxi**: Tongboshan Nature Reserve: SYS a001663–1665, 1667, 1698; Yangjifeng Nature Reserve: SYS a0000317, 0334; Jinggangshan Nature Reserve: SYS a004025–4027; **Zhejiang**: Jingning County: Dongkeng Town: SYS a002725–2726.

*Nidiranadaunchina* (5): **China: Sichuan**: Mt Emei (type locality): SYS a004594–4595; Hejiang County: Zihuai Town: SYS a004930–4932.

*Nidiranahainanensis* (1): **China: Hainan**: Mt Diaoluo (type locality): SYS a003741.

*Nidiranalini* (4): **China: Yunnan**: Jiangcheng County: Hongjiang Town (type locality): SYS a003967–3970.

*Nidirananankunensis* (12): **China: Guangdong**: Mt Nankun (type locality): SYS a003615, 3617–3620, 4019, 4905–4907, 5717–5719 (type series).

*Nidiranapleuraden* (4): **China: Yunnan**: Mt Gaoligong: SYS a003775–3778.
